# Structural basis of impaired disaggregase function in the oxidation-sensitive SKD3 mutant causing 3-methylglutaconic aciduria

**DOI:** 10.1038/s41467-023-37657-9

**Published:** 2023-04-11

**Authors:** Sukyeong Lee, Sang Bum Lee, Nuri Sung, Wendy W. Xu, Changsoo Chang, Hyun-Eui Kim, Andre Catic, Francis T. F. Tsai

**Affiliations:** 1 Verna and Marrs McLean Department of Biochemistry and Molecular Biology, Baylor College of Medicine, Houston, TX 77030 USA; 2 Advanced Technology Core for Macromolecular X-ray Crystallography, Baylor College of Medicine, Houston, TX 77030 USA; 3 Department of Molecular and Cellular Biology, Baylor College of Medicine, Houston, TX 77030 USA; 4 Structural Biology Center, X-ray Science Division, Argonne National Laboratory, Lemont, IL 60439 USA; 5 Department of Integrative Biology and Pharmacology, McGovern Medical School, University of Texas Health Science Center at Houston, Houston, TX USA; 6 Huffington Center on Aging, Baylor College of Medicine, Houston, TX USA; 7 Stem Cells and Regenerative Medicine Center, Baylor College of Medicine, Houston, TX USA; 8 Department of Molecular Virology and Microbiology, Baylor College of Medicine, Houston, TX 77030 USA; 9 Present Address: Louisiana State University Health New Orleans School of Medicine, New Orleans, LA 70112 USA

**Keywords:** X-ray crystallography, Chaperones, Mitochondria

## Abstract

Mitochondria are critical to cellular and organismal health. To prevent damage, mitochondria have evolved protein quality control machines to survey and maintain the mitochondrial proteome. SKD3, also known as CLPB, is a ring-forming, ATP-fueled protein disaggregase essential for preserving mitochondrial integrity and structure. SKD3 deficiency causes 3-methylglutaconic aciduria type VII (MGCA7) and early death in infants, while mutations in the ATPase domain impair protein disaggregation with the observed loss-of-function correlating with disease severity. How mutations in the non-catalytic N-domain cause disease is unknown. Here, we show that the disease-associated N-domain mutation, Y272C, forms an intramolecular disulfide bond with Cys267 and severely impairs SKD3_Y272C_ function under oxidizing conditions and in living cells. While Cys267 and Tyr272 are found in all SKD3 isoforms, isoform-1 features an additional α-helix that may compete with substrate-binding as suggested by crystal structure analyses and in silico modeling, underscoring the importance of the N-domain to SKD3 function.

## Abstract (web summary)

Mitochondrial SKD3 is an essential protein disaggregase. Here, authors solve the X-ray structures of SKD3Ank domain suggesting that the disease-associated mutation Y272C leads to a disulfide bond formation that impairs SKD3 function under oxidizing conditions.

## Introduction

The exigency of all cells to maintain proteostasis requires the ability to survey the proteome and to selectively remove damaged proteins from cells and organelles. Failure of the protein quality control machinery to clear misfolded proteins results in the formation of protein aggregates and other cytotoxic species, which are hallmarks of human disease.

3-methylglutaconic aciduria type VII (MGCA7) is an autosomal recessive, inborn error of metabolism characterized by increased level of 3-methylglutaconic acid. MGCA7 is associated with variable neurologic deficits and neutropenia, which can develop into leukemia and early death in infants. The disease is caused by a deficiency of SKD3^1–4^, an ATP-fueled protein disaggregase^5,6^, which is essential for maintaining mitochondrial cristae morphology^7^. More recently, it was reported that missense mutations in the ATP-binding site of SKD3 are also responsible for severe congenital neutropenia (SCN)^8,9^, an inborn error of granulopoiesis but without 3-methylglutaconic aciduria, suggesting that non-overlapping SKD3 mutations affecting the ATPase activity can be causal for either MGCA7 or SCN. Interestingly, mutations in the non-catalytic N-terminal domain of SKD3 are also associated with MGCA7, but not other SKD3 deficiency-related disorders^8,10^, with the most severe SKD3 variant (SKD3_Y272C_) causing early death in infants^11^. However, the molecular and structural basis of how N-domain mutations cause MGCA7 remains poorly understood.

SKD3 belongs to the ring-forming Hsp100 family that includes mitochondrial ClpX and the microbial ClpB/Hsp104 protein disaggregases^12^. However, SKD3 is absent from microbes and is exclusively found in the mitochondrial intermembrane space (IMS) compartment of vertebrate animal cells^13–15^. SKD3 is targeted to the IMS via an N-terminal leader sequence that is cleaved off by the PARL protease^16^, releasing the mature protein that is a potent protein disaggregase on its own^5^. The mature form of SKD3 can be subdivided into an N-terminal Ankyrin-repeat (Ank) domain of unknown structure and function, and a ring-forming C-terminal nucleotide-binding domain (NBD), which belongs to the HCLR clade of AAA + proteins^12^. Structural studies by cryoEM showed that SKD3 assembles into a nucleotide-stabilized dodecamer composed of two head-to-head hexamers^17–19^. The Ank domains extend vertically from the main body but are poorly resolved in the cryoEM reconstructions, preventing the assignment of secondary structure elements^17,18^.

Here, we present the atomic structures of the isolated Ank domains of SKD3 isoform-1 (ANK_iso1_) and SKD3 isoform-2 (ANK_iso2_). The two isoforms differ by a 30 amino acid stretch present in isoform-1 (exon-5), which represses the protein disaggregating activity of SKD3. Notably, this stretch is flanked by two β-strands that undergo conformational switching and form two α-helices in isoform-2, providing a structural basis for SKD3 activation by substrate. Perhaps more importantly, our structures provide a framework for examining how mutations in the non-catalytic N-domain cause disease. We find that all MGCA7-associated Ank domain mutations cluster to a similar locus, with the clinically most severe SKD3 variant Y272C^11^ forming an intramolecular disulfide bond with Cys267, which impairs protein disaggregation in vitro and displays a loss-of-function phenotype in living cells.

## Results

### The crystal structure of ANK_iso1_ differs from its prediction

**Fig. 1:**
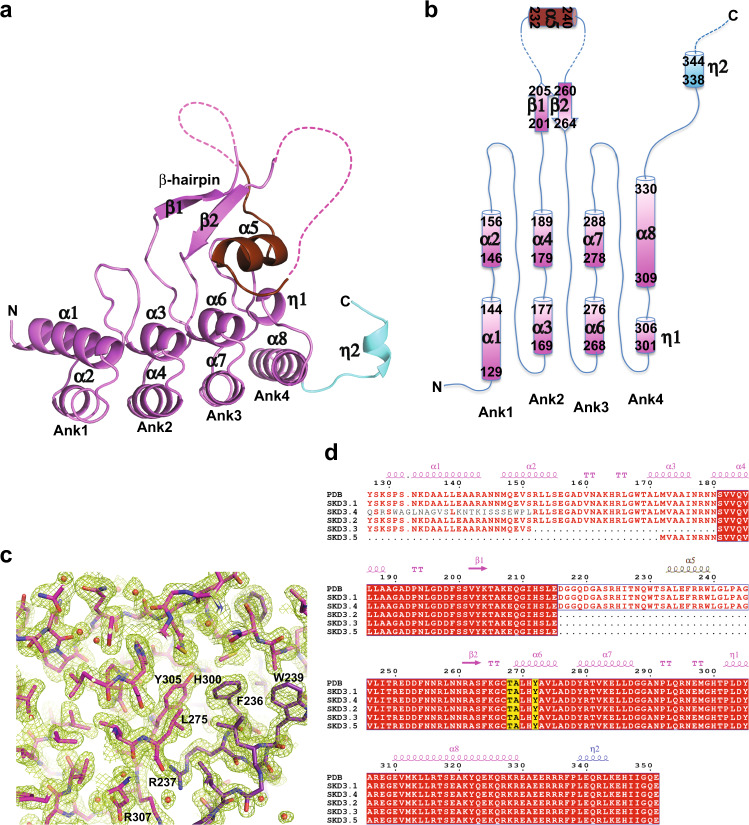
Atomic structure of human ANK_iso1_. **a** Ribbon diagram of the crystallized ANK_iso1_ construct (magenta) comprising three full (Ank1-3) and one degenerate Ank motif (Ank4) and the first 3_10_ helix of the NBD (cyan). The long β-hairpin of Ank2 is labeled with the disordered regions flanking helix α5 (brown) indicated by dashed lines. The same color scheme is used in all figure panels **b** Topology diagram of human ANK_iso1_. **c** Section of the simulated-annealing 2Fo-Fc composite omit map (green mesh) contoured at the 1.0 σ level. **d** Sequence alignment of the Ank domain of SKD3 isoforms. Conserved residues are highlighted red, and residues mutated in MGCA7 patients are highlighted yellow. SKD3 isoform-1 and isoform-2 differ by a 30 amino acid stretch in the Ank domain (residues 216-245) but are otherwise identical in amino acid sequence. Secondary structure elements are shown with helix α5 colored brown and the first 3_10_ helix (η2) of the NBD colored blue. “TT” indicates a strict β-turn. Figure was generated using ESPript 3.0^39^.

Owing to a lack in our molecular understanding of the Ank domain, we solved the crystal structure of ANK_iso1_ at 1.81-Å resolution using the single wavelength anomalous diffraction (SAD) technique (Supplementary Table 1). Although the crystal structure contains only one ANK_iso1_ molecule in the crystallographic asymmetric unit, it appears to form a dimer in solution (Supplementary Fig. 1a). The ANK_iso1_ structure consists of three canonical Ank motifs (Ank1-3) and a degenerated fourth Ank motif (Ank4). While Ank1 to Ank3 share the helix-turn-helix-hairpin-loop motif, Ank4 lacks the hairpin and features a 3_10_ helix (η1) at the first position (Fig. 1a-b). Amongst the Ank motifs, Ank2 is most unusual and features a long β-hairpin-helix motif instead of the canonical loop. The electron density for helix α5 (residues 232-240) is clearly visible in our experimentally phased map, which is sandwiched between the β-hairpin and the concave surface of the Ank domain (Fig. 1a). The interaction is mediated by hydrophobic contacts between the side chains of Phe236, Arg237 and Trp239 of α5 and His300 as well as Tyr305 from η1 (Fig. 1c). In the crystal, this sandwich conformation is stabilized by the C-terminal segment of a symmetry-related neighboring Ank domain, which wraps around the β-hairpin, and may provide a structural basis for dimer formation in solution (Supplementary Fig. 2). Although the overall structure of ANK_iso1_ resembles the AlphaFold2 model (AF-Q9H078-F1), we note that the long β-hairpin with the intervening α5 helix is unexpected and markedly differs in both the location and secondary structure from the predicted AlphaFold2 model (Supplementary Fig. 3).

### The Ank domain of SKD3 is a polypeptide binding domain

**Fig. 2:**
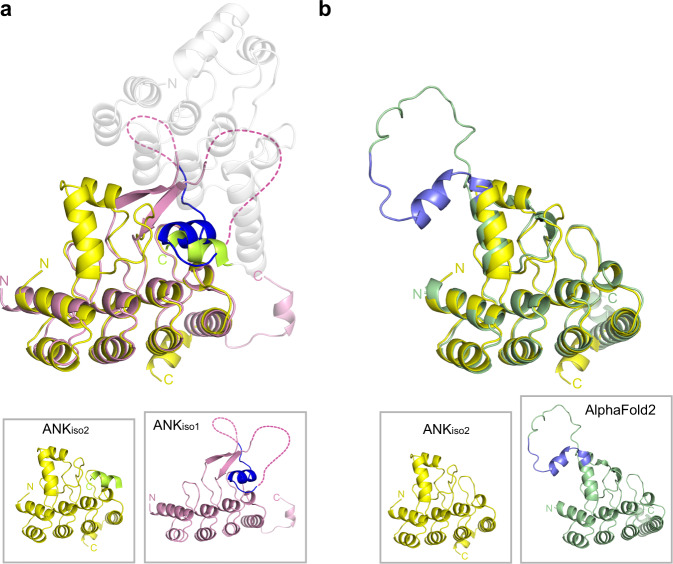
The crystal structure of human ANK_iso2_ differs from ANK_iso1_ and is similar to the AlphaFold2 model. **a** Superposition of the crystal structures of ANK_iso2_ (yellow) and ANK_iso1_ (magenta). The superposition shows the location of the long β-hairpin-helix motif, including helix α5 (blue), which is only found in ANK_iso1_. It is entirely fortuitous that the C-terminal His_6_-tag (light green) of a crystal symmetry-related neighboring Ank domain (gray) is bound in *trans* to ANK_iso2_ (yellow). The N- and C-termini are labeled. The figure shows that the His_6_-tag binds to the same concave surface that is occupied by helix α5 (blue) in the ANK_iso1_ structure. **b** Superposition of the crystal structure of ANK_iso2_ (yellow) and the predicted AlphaFold2 model (AF-Q9H078-F1, green) of ANK_iso1_ with amino acid residues 228-244 in blue.

Could the predicted AlphaFold2 model be incorrect? Several alternatively spliced SKD3 variants exist, which group into one of two main isoforms (Fig. 1d). SKD3 isoform-1 (i.e., SKD3) differs from SKD3 isoform-2 (SKD3_iso2_) by a 30 amino acid stretch in the Ank domain, which includes helix α5 but is otherwise identical in amino acid sequence. After solving the atomic structure of ANK_iso1_, we determined the 1.65-Å resolution crystal structure of ANK_iso2_ (Supplementary Table 1). ANK_iso2_ crystallized as a monomer and is a monomer in solution (Supplementary Fig. 1b). However, to our surprise, the structure of ANK_iso2_ markedly differs from the structure of ANK_iso1_ and is entirely α-helical with the long β-hairpin undergoing a conformational switch (Fig. 2a). Unlike ANK_iso1_, the crystal structure of ANK_iso2_ closely resembles the predicted AlphaFold2 model (Fig. 2b and Supplementary Fig. 3) and superimposes with a root mean square deviation of only 0.541 Å over 143 Cα atoms. Notably, the concave surface that was previously occupied by the α5 helix (Fig. 1a) is now bound by the C-terminal helix of a symmetry-related neighboring ANK_iso2_ domain (Fig. 2a). This interaction is totally fortuitous and could mimic an interaction with substrate that has displaced helix α5.

Ankyrin repeats are one of the most abundant motifs found in eukaryotic proteins and the Ank domain is the defining feature that distinguishes mammalian SKD3 from microbial Hsp100 unfoldases. Despite their different architecture, it was recently shown that SKD3 is a protein disaggregase that acts on several model substrates including aggregated firefly luciferase (FFL) and α-synuclein fibrils^5^. It is known that Ank domain-containing proteins often bind substrates via their concave surface formed by the inner helices and hairpin loops^20^. Superposing the crystal structure of ANK_iso1_ (Supplementary Fig. 4a) onto that of the unrelated but structurally homologous Kidney ankyrin repeat-containing protein, KANK1, bound to a segment of the KANK1-binding partner, KIF21A^21^, (Supplementary Fig. 4b) shows a helical segment of the KIF21A peptide overlapping with helix α5 (Supplementary Fig. 4c), further substantiating that the SKD3 Ank domain is involved in substrate binding.

### SKD3 isoforms are functional protein disaggregases

**Fig. 3:**
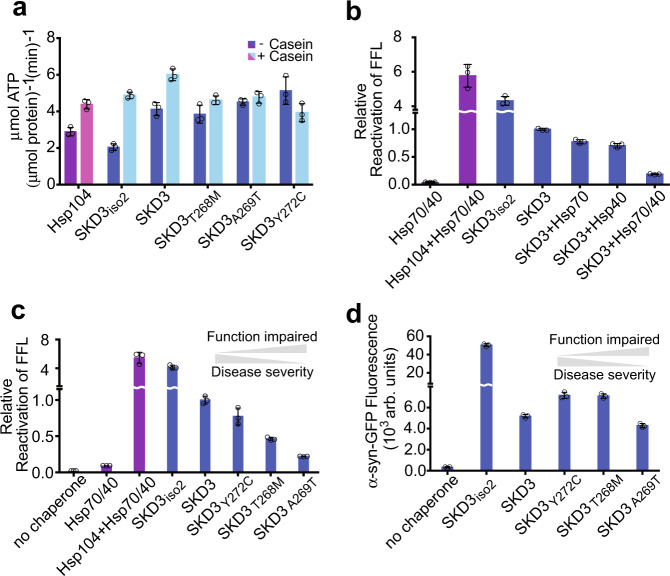
MGCA7-associated SKD3 variants are functional protein disaggregases. Bar graphs show averages of three independent measurements (*n* = 3) ±SD. Source data are provided as a Source Data file. **a** ATPase activities of Hsp104 (purple bars), and SKD3 and SKD3 variants (blue bars) without and with κ-casein stimulation as indicated by the dark and light hues. **b** Mitochondrial SKD3 is a stand-alone protein disaggregase that is inhibited by cytosolic Hsp70 and Hsp40 chaperones. The figure shows the relative reactivation of firefly luciferase (FFL) from aggregates by the indicated protein disaggregase and chaperone systems. **c**, **d** SKD3 Ank domain variants are functional protein disaggregases under reducing conditions. Protein disaggregating activities of SKD3 and SKD3 Ank domain variants (blue bars) were measured using (**c**) amorphous FFL aggregates and (**d**) α-syn fibrils. The protein disaggregating activity of the Hsp104 bi-chaperone system with FFL is shown for comparison (purple bars). The data reveal an inverse relationship (gray arrows) between functional impairment of SKD3 mutants in protein disaggregation and disease severity associated with these mutants in MGCA7 patients^11^.

The structure of ANK_iso1_ suggested that binding of helix α5 may compete with substrate interaction. Because SKD3 isoform-2 (SKD3_iso2_), which lacks helix α5, is the predominant SKD3 isoform in HEK-293T cells^9,18^, we wished to determine whether this helix inhibits SKD3 function. If so, we expect that deleting helix α5 enhances the SKD3 chaperone activity. To test this, we bacterially expressed and purified the mature forms of SKD3 and SKD3_iso2_. As anticipated, both SKD3 isoforms are functional ATPases, whose ATPase activity is stimulated by casein (Fig. 3a). Although the basal ATPase activity of SKD3_iso2_ is reduced by half compared to isoform-1, we found that the ability of SKD3_iso2_ to recover functional protein from amorphous FFL aggregates is >4-fold higher than that of SKD3, and comparable to levels observed with the more powerful Hsp104 bi-chaperone system (Fig. 3b), indicating that helix α5 may indeed repress the protein disaggregating activity of isoform-1. However, contrary to Hsp104, SKD3’s ability to facilitate protein disaggregation was impaired in the presence of cytosolic Hsp70 and Hsp40, with a greater combined effect with Hsp70/Hsp40 than the expected sum of inhibition with each chaperone alone (Fig. 3b). Because the IMS compartment does not harbor homologs of cytosolic Hsp70 or Hsp40 that may compete with SKD3 for binding to aggregated proteins, synergistic inhibition of SKD3 by cytosolic chaperones could provide a mechanism to prevent SKD3 function outside of mitochondria.

### MGCA7-associated ANK mutants are functional disaggregases

Next, we wished to determine the molecular basis of how patient-derived missense mutations in the Ank domain cause MGCA7 and early death in infants^11^. It was previously proposed that a loss of protein disaggregating activity resulting from mutations in the catalytic NBD correlated with disease severity^5,8^. To test whether missense mutations in the non-catalytic Ank domain follow this corollary, we generated the T268M, A269T, and Y272C single mutants found in MGCA7 patients^3,4,22^, with the Y272C variant being the most severe^11^. We introduced our Ank domain mutants into isoform-1 that was also used by others for their biochemical studies^5,6,8,17,19^. However, other isoforms such as isoform-2 are expressed in different cell types^9,18^ and most tissues (https://gtexportal.org/home/gene/CLPB), and which isoform is more disease relevant is not entirely clear. However, the only confirmed report of a patient-derived MGCA7 mutation (T268M) was found in isoform-1^3^, underscoring the importance of analyzing Ank domain mutations in this protein background.

All three Ank domain mutants are functional ATPases (Fig. 3a) and form higher order oligomers that are indistinguishable from SKD3 (Supplementary Fig. 5). To measure their protein disaggregating activity, we used two substrates, amorphous FFL aggregates (Fig. 3c) and fibrillar α-synuclein-GFP (α-syn) (Fig. 3d), which differ in aggregate structure and amino acid composition to avoid unintentional bias. Interestingly, SKD3_Y272C_ that showed the highest basal ATPase activity in our panel (Fig. 3a), displayed a moderate protein disaggregating activity with both substrates, which was higher than other Ank domain mutants (Fig. 3c) and even exceeded the level observed with SKD3 (Fig. 3d), but was always lower than SKD3_iso2_ (Fig. 3c, d). Notably, we found that the protein disaggregating activity of SKD3_iso2_ was nearly 10-fold higher than that of SKD3 using α-syn fibrils as substrate (Fig. 3d), indicating that SKD3 displays a substrate preference. However, most importantly, we did not observe a direct correlation between loss of protein disaggregating activity by SKD3 mutants (Fig. 3c, d) and their associated disease severity in MGCA7 patients^11^. Instead, we observed an inverse relationship amongst SKD3 mutants with the clinically most severe Y272C variant being the least functionally impaired under our standard assay condition with DTT (Fig. 3c, d).

**Fig. 4:**
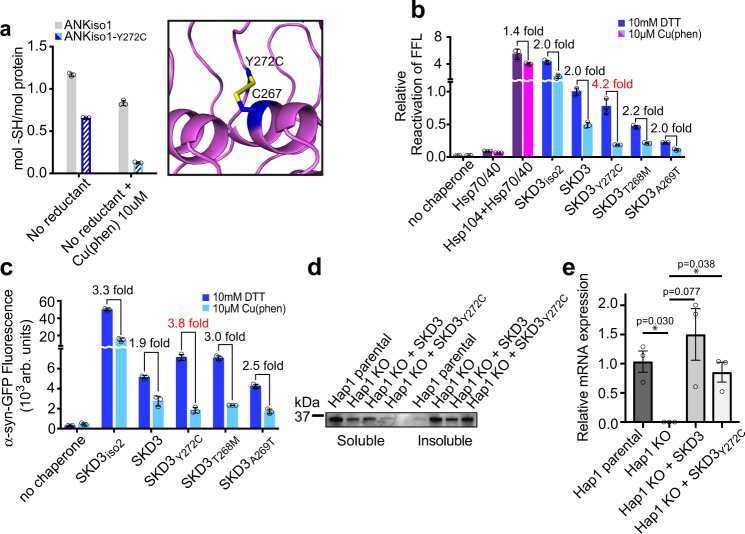
SKD3 is an oxidation-sensitive protein disaggregase. Bar graphs show averages of three independent measurements (*n* = 3) ±SD. Source data are provided as a Source Data file. **a** ANK_iso1-Y272C_ (colored bars) but not ANK_iso1_ (gray bars) forms an intramolecular disulfide bond under oxidizing condition as confirmed by using Ellman’s reagent. The predicted structure of an intramolecular disulfide bonded SKD3_Y272C_ variant is shown. **b**, **c** The MGCA7-associated Y272C mutation results in a major loss of protein disaggregating activity but only under oxidizing conditions. Protein disaggregating activities by the indicated chaperones were measured using (**b**) amorphous FFL aggregates and (**c**) α-syn fibrils under reducing conditions with DTT (purple and blue) or under catalyzed oxidizing conditions with Cu(phen) and in the absence of reductant (magenta and cyan). **d** Representative western blot of a sedimentation assay showing the relative solubility of HAX1 protein in the supernatant (soluble) and pellet fraction (insoluble) of lysed mitochondria isolated from wild-type (parental) and *SKD3* knockout (KO) Hap1 cells. HAX1 solubility is partially restored by viral transfection of *SKD3* wild-type, but not by *SKD3*_*Y272C*_ mutant (*n* = 3). **e** Viral transfection of *SKD3* or *SKD3*_*Y272C*_ into *SKD3* knockout Hap1 cells was confirmed by RT-qPCR. P-values were calculated with the two-tailed paired Student’s *t*-test (*p* < 0.05 = *).

The crystal structures of the Ank domain showed that all three MGCA7-associated missense mutations map to the Ank2 motif and cluster around Cys267 that follows the long β-hairpin. Although SKD3 features as many as nine cysteines in its primary sequence, only one (Cys267) is found in the Ank domain. Interestingly, the Cα distance between Cys267 and Tyr272 is 7.0-Å, which is within the range of Cα distances between disulfide bonded cysteines observed in protein structures^23^. Indeed, modeling the Y272C variant in silico showed that Y272C potentially forms an intramolecular disulfide bond with Cys267 (Fig. 4a), which we confirmed biochemically by using Ellman’s reagent (Fig. 4a). Because the IMS compartment is a highly oxidizing environment^24^, we directly compared the activity of our SKD3 variants under reducing and oxidizing conditions (Fig. 4b, c). Unsurprisingly, all chaperone proteins including the Hsp104 bi-chaperone system are sensitive to oxidation and displayed a lower protein disaggregating activity in the presence of copper phenanthroline (Cu(phen)), a strong oxidant, but generally followed the same trend as observed under reducing conditions (Fig. 4b, c). However, strikingly, we found that SKD3_Y272C_, which showed a mildly stimulated basal ATPase activity under reducing conditions (Fig. 3a), was most severely impaired under oxidizing conditions and displayed a 4.2-fold reduction in protein disaggregation with aggregated FFL (Fig. 4b) and a 3.8-fold reduction with α-syn fibrils (Fig. 4c). This reduction in protein disaggregating activity by SKD3_Y272C_ is significantly greater than the average drop observed with the Hsp104 bi-chaperone system and other SKD3 mutants in our panel and is at least 2-fold greater than observed with SKD3 (Fig. 4b, c). Because α-synuclein is a cysteine-free protein, we ruled out irreversible damage of substrate as a cause of reduced protein disaggregation. We reasoned that formation of an intramolecular disulfide bond between Cys267 and Y272C renders SKD3_Y272C_ more sensitive to oxidation and provides a structural basis for the greater loss in protein function. Consistent with this notion, the T268M and A269T mutants, which map to a similar locus but cannot form a disulfide bond, display a less severe disease phenotype^11^.

### SKD3_Y272C_ is defective and cannot rescue a *SKD3* knockout

Next, we wished to determine whether SKD3_Y272C_ displays a loss of protein function in human cells. It was previously shown that SKD3 maintains solubility of HAX1^5^, an omnipresent protein associated with severe congenital neutropenia in patients^9,25^. As previously reported, we find that a *SKD3* knockout results in massive protein aggregation with HAX1 accumulating in the insoluble mitochondrial pellet (Fig. 4d and Supplementary Fig. 6). If the Y272C mutation causes a loss of SKD3 function, we reasoned that viral transfection with *SKD3* wild-type but not with *SKD3*_*Y272C*_ could restore HAX1 solubility in *SKD3* knockout Hap1 cells. Indeed, we find that viral transfection with *SKD3* reduced the level of aggregated HAX1 in the insoluble mitochondrial fraction, whereas viral transfection with *SKD3*_*Y272C*_ did not (Fig. 4d and Supplementary Fig. 6). Unexpectedly, we observed a further depletion of soluble HAX1 following viral transfection with *SKD3*_*Y272C*_ compared to *SKD3* knockout Hap1 cells (Fig. 4d and Supplementary Fig. 6). While the nature of this depletion remains unknown, and we cannot exclude a potential loss of protein stability or defects in mitochondrial import induced by the mutation, we confirmed that both wild-type and mutant *SKD3* were expressed at similar mRNA levels following viral transfection (Fig. 4e).

## Discussion

Our crystal structures of ANK_iso1_ and ANK_iso2_ provide a molecular framework of how mutations in the non-catalytic Ank domain cause MGCA7. It highlights the heightened oxidation sensitivity of the patient-derived Y272C mutant and the regulatory function of helix α5 in repressing the protein disaggregating function of isoform-1. It is widely appreciated that many proteins are sensitive to oxidative stress and molecular chaperones are no exception. However, we find that the loss in protein disaggregating activity by SKD3_Y272C_ is significantly greater than the average loss in protein disaggregation observed for SKD3 and other SKD3 variants following oxidative challenge using two different substrates. We reason that an intramolecular disulfide bond between Cys267 and Y272C provides a structural basis for disease pathomechanism and is compatible with the loss of function phenotype of SKD3_Y272C_ in living cells.

How mutations in SKD3 cause different diseases is poorly understood. This is further complicated by the complex recessive nature of MGCA7-linked mutations, what isoform is found in what tissue, and which isoform is relevant for disease. Although the vast majority of disease mutations were identified by exome sequencing, it was reported that one patient-derived Ank domain mutation (T268M) is found in isoform-1^3^, supporting the significance of this work. Previously, it was proposed that mutations near the ATP-binding pocket, which inhibit SKD3 function, are responsible for SCN^8^ and MGCA7^5^. However, SCN and MGCA7 are caused by different mutations that are unique to each disorder indicating that abolishing ATPase function as the basis for disease may be an oversimplification.

It was recently reported that SKD3 is a dodecamer when bound to casein, which is an unfolded polypeptide^17,18^. We note that the unusual architecture of the SKD3 dodecamer composed of two head-to-head hexamers would preclude binding of an aggregated protein via the N-domain. Although dodecamers of head-to-head hexamers have been reported for other ATP-dependent protein unfoldases^26–28^, they are either functionally repressed^27^ or only partially active^26^, suggesting that the ring-forming hexamer is the physiologically active conformation^27,29,30^. Whether protein disaggregation requires SKD3 to be a dodecamer or a hexamer like all other Hsp100 unfoldases remains to be determined. To our knowledge, the finding that SKD3_Y272C_ is sensitive to oxidation and forms a non-native disulfide bond has not previously been reported, and highlights the importance of biochemical processes in the IMS compartment that are essential for maintaining mitochondrial structure and function.

## Methods

### Protein production

Human *SKD3* (residues 127-707), *SKD3* mutants, and *ANK*_*iso1*_ (residues 132-351) were cloned into a modified pProEX-HTb vector, which adds a Tobacco Etch Virus (TEV) protease cleavable N-terminal His_6_-SUMO-tag. *ANK*_*iso2*_ (residues 127-302), which lacks residues 216-245 of isoform-1, was cloned into the pET-28b(+) plasmid (MilliporeSigma), which adds a non-cleavable C-terminal His_6_-tag. All *SKD3* mutants were commercially synthesized (GenScript USA, Piscataway, NJ) and introduced into the MGCA7 disease-relevant isoform-1 background. Transformed *Escherichia coli* BL21-CodonPlus (DE3)-RIL cells (Cat. #230245, Agilent Technologies) were cultured at 37 °C in LB medium supplemented with 34 μg/mL chloramphenicol and either 100 μg/mL ampicillin (pProEX) or 50 μg/mL kanamycin (pET) until mid-log phase, induced with 0.3 mM IPTG, and continued to grow at 18 °C for an additional 16 h before harvesting. Cells were suspended in 25 mM Tris-HCl pH 8.8 (SKD3) or pH 8.5 (ANK_iso1_ and ANK_iso2_), 0.3 M NaCl, 30 mM imidazole, 5% glycerol, and 5 mM β-mercaptoethanol (β-ME) supplemented with 1 mM phenylmethylsulfonyl fluoride (PMSF), 1 mM benzamidine, and one tablet of cOmplete protease inhibitor cocktail (Cat. # 11873580001, MilliporeSigma), and were lysed on ice using a microfluidizer processor (Microfluidics International Corporation, Westwood, MA). His-tagged proteins were purified from the soluble lysate using immobilized metal chelate affinity chromatography (IMAC). When applicable, the His-tag was cleaved off with His_6_-TEV protease and reapplied to an IMAC column to remove the liberated His-tag, the TEV protease, and any uncleaved His-tagged protein. SKD3, SDK3 variants, as well as ANK_iso1_ and ANK_iso1-Y272C_ were further purified by anion exchange and size-exclusion chromatography (SEC) for biochemical assays. Hsp104^31^, human Hsp70^32^, and Hsp40 (Ydj1)^33^ were bacterially overexpressed and purified using IMAC followed by anion exchange (Hsp104 and Hsp70) or hydrophobic interaction and anion exchange chromatography (Hsp40). For crystallization, ANK_iso1_ and ANK_iso2_ were purified by IMAC and anion exchange chromatography, and ANK_iso1_ was further purified using a Toyopearl Butyl-650 chromatography column (Tosoh Bioscience LLC, King of Prussia, PA). Se-Met substituted ANK_iso1_ (residues 132-351) was prepared by transforming *E. coli* BL21-CodonPlus (DE3)-RIL-X (Cat. #230265, Agilent Technologies) with pANK_iso1_ in the presence of the appropriate antibiotics and overexpressing the protein in defined medium supplemented with 50 mg/L seleno-DL-methionine. Preparation of Se-Met ANK_iso1_ was otherwise identical to the native protein.

To make α-syn fibrils, α-synuclein-GFP was bacterially expressed from pRK172-α-syn-TEV-GFP (Plasmid #166671, Addgene), which adds a His_6_-tag and a TEV cleavage site between mouse α-synuclein and GFP^34^. In brief, transformed *E. coli* BL21-CodonPlus (DE3)-RIL cells (Cat. #230245, Agilent Technologies) were cultured at 37 °C in terrific broth medium supplemented with 100 μg/mL ampicillin and 34 μg/mL chloramphenicol. Cells were induced at mid-log phase with 0.3 mM IPTG and continued to grow at 18 °C for an additional 18 h. The cell pellet was suspended in 25 mM Tris-HCl pH 8.0, 0.5 M NaCl, 30 mM imidazole, 5 mM β-ME, 1 mM PMSF, 1 mM benzamidine, and one tablet of cOmplete protease inhibitor cocktail (Cat. # 11873580001, MilliporeSigma) and lysed on ice using a microfluidizer processor (Microfluidics International Corporation, Westwood, MA). α-synuclein-GFP was purified from the soluble lysate by IMAC followed by anion exchange chromatography. α-synuclein-GFP seeds were generated by concentrating the protein to 170 μM (monomer) in 25 mM Tris-HCl pH 8.0 and 5 mM β-ME, followed by incubation with shaking at 37 °C for 4 days. The turbid sample was vortexed and used to seed fiber formation of a freshly purified α-synuclein-GFP sample (1.62 mM, monomer). This sample became turbid overnight and was placed in a shaking incubator at 37 °C for 4 days before being centrifuged three times at 20,000 x g for 20 min at 4 °C with removal of the supernatant after each cycle to deplete all soluble α-synuclein-GFP. The final sample was vortexed vigorously to obtain a homogeneous mixture and distributed into 50 μL aliquots that were flash frozen and stored at −80 °C.

### Protein crystallization

Native ANK_iso1_ (40 mg/mL) and ANK_iso2_ (18 mg/mL) or Se-Met substituted ANK_iso1_ (35 mg/mL) in 25 mM Tris-HCl pH 8.5, 100 mM NaCl, and 1 mM TCEP were crystallized at 14 °C using the hanging drop vapor diffusion method by mixing 0.7 μL protein solution with an equal volume of precipitant consisting of 50 mM EPPS pH 8.0, 35% (v/v) PEP426, and 250 mM ammonium sulfate (native ANK_iso1_); 50 mM MES pH 6.5, and 35% (v/v) PEP426 (Se-Met ANK_iso1_); and 100 mM Bis-Tris propane pH 9.0, 4 M potassium formate, and 2% (w/v) mPEG 2000 (ANK_iso2_). Crystals belonged to the primitive monoclinic space group, *P* 2_1_ (ANK_iso1_) or *P* 6_5_22 (ANK_iso2_), with one molecule in the asymmetric unit. For data collection, crystals were harvested in reservoir solution supplemented with 20% (v/v) glycerol for native ANK_iso1_, 12.5% (v/v) glycerol for Se-Me ANK_iso1_, and 15% (v/v) glycerol for ANK_iso2_ crystals, and flash frozen in liquid nitrogen.

### Structure determination

A 1.95-Å resolution SAD data set near the selenium absorption peak (0.9794 Å) for ANK_iso1_, and a native data sets at 1.81-Å (ANK_iso1_) and 1.65-Å resolution (ANK_iso2_) were collected at 100 K using a Pilatus 3X 6 M detector and the SBCCollect control software at the APS SBC ID19 beamline of Argonne National Laboratory (Lemont, IL). Data were processed using HKL3000^35^ (ANK_iso1_) or DIALS^36^ (ANK_iso2_), and SAD phasing, density modification (DM), and initial model building were done using the structure module of the HKL3000 software suite^35^. A total of six selenium sites were found and initial phases were calculated using MLPHARE yielding a mean Figure of Merit of 0.138 for data between 45.67–1.95 Å resolution, which was further improved to 0.74 after DM. The model was built in COOT^37^ and refined using PHENIX^38^. The crystal structure of ANK_iso2_ was subsequently determined by the molecular replacement technique using the structure of ANK_iso1_ as a search model. The refined structures of ANK_iso1_ and ANK_iso2_ have excellent stereochemical properties with 100.0% (ANK_iso1_) and 98.9% of residues (ANK_iso2_) in the Ramachandran favored region.

### ATPase activity assay

The ATPase activity of mature SKD3 and variants (0.5 µM monomer) was determined at 25 °C in 25 mM HEPES-KOH pH 8.0, 150 mM potassium acetate, 10 mM magnesium acetate, 10 mM DTT, and 2 mM ATP using the malachite green colorimetric assay. The reaction was allowed to proceed for 12 min before quantifying the amount of inorganic phosphate by mixing 10 µL of reaction sample with 160 µL of malachite green assay solution (0.03 % malachite green, 1.05 % ammonium molybdate, 1 N HCl, 0.02 % Triton X-100). After incubating the mixture for 1 min at room temperature, 20 µL of 34 % citric acid was added to stop the reaction followed by measuring the absorbance at 660 nm using a Spark microplate reader (Tecan US, Morrisville, NC). The specific ATPase activity was calculated after subtracting the amount of inorganic phosphate in the absence of protein.

### Protein disaggregation assay

Recombinant firefly luciferase (FFL; 50 µM) (Cat. #E1701, Promega) was denatured using 8 M urea in refolding buffer (25 mM HEPES-KOH pH 8.0, 150 mM potassium acetate, 10 mM magnesium acetate, 10 mM DTT) at 30 °C for 30 min, rapidly diluted 100-fold in refolding buffer, and snap frozen in liquid nitrogen. FFL aggregates (50 nM final, monomer) were mixed with 1 μM (final, monomer) SKD3 and incubated with an ATP regenerating system (1 mM creatine phosphate, 0.25 µM creatine kinase, and 5 mM ATP) in refolding buffer at 30 °C for 90 min. Luminescence was measured in a 96 well plate (Cat. #655209, Greiner Bio-One) using a Spark microplate reader (Tecan US, Morrisville, NC). To measure the protein disaggregating activity under oxidizing condition, the assay was performed in refolding buffer with 10 μM Cu(phen) and in the absence of reductant. Data were normalized by dividing the recovered FFL activity in the presence of the indicated chaperones by the recovered activity with SKD3 in refolding buffer with DTT.

For the fiber disaggregation assay, α-syn fibrils were thawed and spun down three times at 20,000 x g for 20 min at 4 °C. To ensure that no soluble α-synuclein-GFP was present due to spontaneous resolubilization, the supernatant was removed after each cycle and fibrils were resuspended in refolding buffer. For the assay, α-syn fibrils (~2 μM final, monomer) were mixed with 5 μM (final, monomer) SKD3 and incubated with an ATP regenerating system (10 mM creatine phosphate, 1 µM creatine kinase, and 10 mM ATP) in refolding buffer at 30 °C for 90 min. After incubation, the samples were spun down once (20,000 x g, 20 min, 4 °C), and the supernatant was pipetted out and diluted twice in refolding buffer before measuring the fluorescence signal (488 nm excitation, 510 nm emission, 7.5 nm bandwidth) in a 384-black well plate (Cat. #3544, Corning) using a Tecan Spark microplate reader (Tecan US, Morrisville, NC). Measurements under oxidizing conditions were performed in refolding buffer with 10 μM Cu(phen) and in the absence of reductant.

### Disulfide crosslinking

The isolated Ank domain of SKD3 (ANK_iso1_) and SKD3_Y272C_ (ANK_iso1-Y272C_) were buffer exchanged into 25 mM Tris-HCl pH 8.5 and 200 mM NaCl using a Superdex 75 10/300 GL size-exclusion chromatography column (Cytiva) to remove any residual reductant. Proteins (150 μM) were incubated at room temperature for 10 min in the presence of 10 μM Cu(phen) to catalyze disulfide bond formation. Proteins were denatured in 6 M guanidine hydrochloride to expose any free sulfhydryl groups prior to adding Ellman’s reagent (0.18 mM final), followed by incubating the mixture at 25 °C for 15 mins. Free sulfhydryl groups were quantified by measuring the absorbance at 412 nm in a 384-well black plate (Cat. #3544, Corning) using a TECAN Spark microplate reader (Tecan US, Morrisville, NC).

### Tissue culture and viral transfection

Isogenic Hap1 parental (Cat. #C631) and Hap1 *SKD3* knockout cells (Cat. #HZGHC007326c001) were directly acquired from Horizon Discovery Biosciences Ltd. (Cambridge, UK) and grown in IMDM (Cat. #12440053, ThermoFisher Scientific) with 10% FCS and 1% penicillin-streptomycin at 37 °C in a humidified tissue culture incubator (5% CO_2_). Cells were passaged for <30 passages and propagated following dissociation with 0.25% trypsin.

Full-length *SKD3* and *SKD3*_*Y272C*_ were cloned into the lentiviral pLVX-Puro vector (Cat. #632164, Takara) using uniquely engineered 5' *XhoI* and 3' *BamHI* restriction sites and confirmed by nanopore sequencing. Hap1 *SKD3* knockout cells were transduced with virus encoding pSKD3 or pSKD3_Y272C_ and selected with puromycin (1.5 μg/mL). Gene expression was confirmed by RT-qPCR (Cat. #11736-059, ThermoFisher Scientific) using the following primers: *SKD3* (Fwd: CAGCAAGAGTCCGTCCAACAA; Rev: GCCAAGTCTGTGCTTTGCATT) and *GAPDH* (Fwd: GGAGCGAGATCCCTCCAAAAT; Rev: GGCTGTTGTCATACTTCTCATGG). RNA was extracted from 2.5 million Hap1 cells (Cat. #74134, Qiagen).

### Mitochondria isolation

Cells were harvested following 0.25% trypsin dissociation, quenched in full medium, and washed in PBS prior to cell counting using a Cellometer Auto 2000 Cell Viability Counter (Nexcelom Bioscience, Lawrence, MA). Batches of equal cell count were aliquoted (2-4 million per experiment), and cell viability was confirmed >90% through AOPI double staining with ViaStain (Cat. #CS2-106, Nexcelom Bioscience). Mitochondria isolation, protein quantification, and enrichment of soluble and insoluble mitochondrial fractions were performed as previously described^5^ following manual lysis using a Dounce homogenizer on ice (40 strokes per cell type in 0.8 mL SM buffer consisting of 50 mM Tris-HCl pH 7.4, 0.25 M sucrose, 2 mM EDTA, and 1% BSA). Equal volumes of mitochondrial fractions were examined by western blot (Cat. #4568123 and #1704156, Bio-Rad Laboratories) and probed with 1:2,000 anti-HAX1 antibody (Cat. #ab137613 and #ab6721, Abcam) in 1% milk/TBST overnight after blocking in 5% milk/TBST.

### Reporting summary

Further information on research design is available in the Nature Portfolio Reporting Summary linked to this article.

## Supplementary information

Supplementary Information

Reporting Summary

## Data Availability

Atomic coordinates and accompanying structure factors in this study have been deposited in the RCSB under accession codes PDB: 8DEH (ANK_iso1_) and PDB: 8FDS (ANK_iso2_). Source data are provided with this paper.

## Source data

Source Data
