# Supplementary material for: Structural basis of impaired disaggregase function in the oxidation-sensitive SKD3 mutant causing 3-methylglutaconic aciduria
Source: Nat Commun. 2023 Apr 11;14:2028. doi: 10.1038/s41467-023-37657-9 (PMC10090083; doi:10.1038/s41467-023-37657-9)
Supplement: Supplementary file 1 — Supplementary Information [file 41467_2023_37657_MOESM1_ESM.pdf]

SUPPLEMENTARY INFORMATION

Structural Basis of Impaired Disaggregase Function in the Oxidation-sensitive SKD3 Mutant Causing 3-Methylglutaconic Aciduria

Sukyeong Lee[1,2], Sang Bum Lee[1,2], Nuri Sung[1,3], Wendy W. Xu[2§], Changsoo Chang[4], Hyun-Eui Kim[5], Andre Catic[3,6,7], and Francis T.F. Tsai[1,2,3,8*]

[1] Verna and Marrs McLean Department of Biochemistry and Molecular Biology, Baylor College of Medicine, Houston, Texas 77030, USA, [2] Advanced Technology Core for Macromolecular X-ray Crystallography, Baylor College of Medicine, Houston, Texas 77030, USA, [3] Department of Molecular and Cellular Biology, Baylor College of Medicine, Houston, Texas 77030, USA, [4] Structural Biology Center, X-ray Science Division, Argonne National Laboratory, Lemont, IL 60439, USA, [5] Department of Integrative Biology and Pharmacology, McGovern Medical School, University of Texas Health Science Center at Houston, Houston, TX, USA, [6] Huffington Center on Aging, Baylor College of Medicine, Houston, TX, USA, [7] Stem Cells and Regenerative Medicine Center, Baylor College of Medicine, Houston, TX, USA, [8] Department of Molecular Virology and Microbiology, Baylor College of Medicine, Houston, Texas 77030, USA.

[§]Present address: Louisiana State University Health New Orleans School of Medicine, New Orleans, LA 70112, USA.

[*]Correspondence: ftsai@bcm.edu

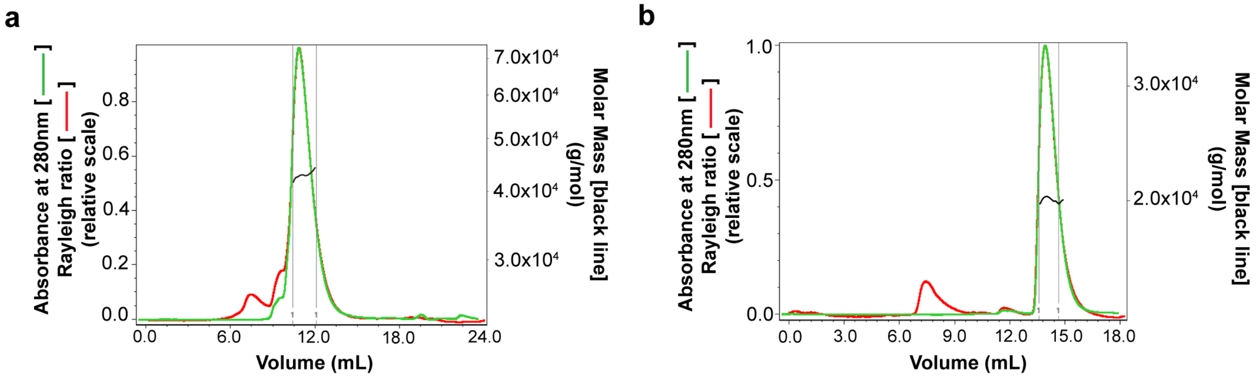

Supplementary Fig. 1: Analysis of the oligomeric state of the isolated Ank domains in solution. The oligomeric state was determined by SEC-MALS using a Superdex 75 Increase 10/300 GL column. **(a)** The SEC-MALS analysis suggests that ANK_{iso1} is a dimer. **(b)** The SEC-MALS analysis suggests that ANK_{iso2} is a monomer.

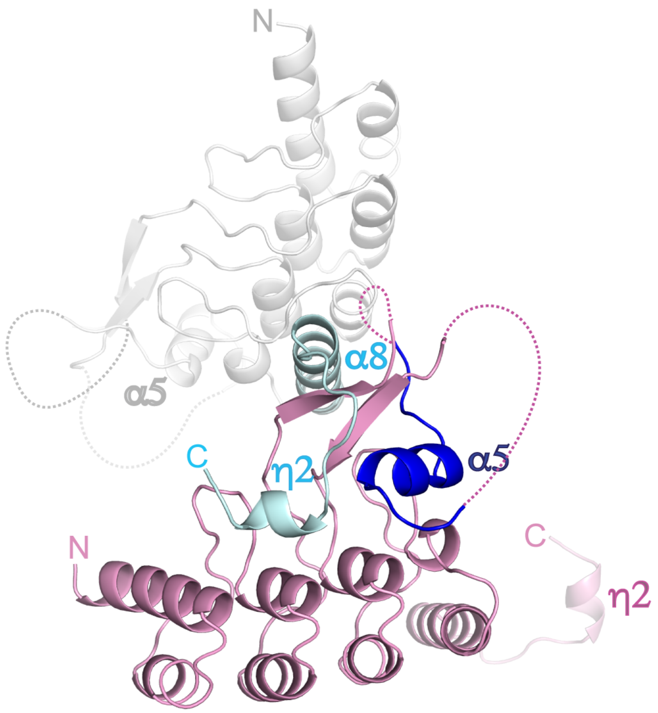

Supplementary Fig. 2: Stabilization of the repressed conformation of ANK_{iso1} as seen in the crystal structure. Ribbon diagram of the X-ray structure of ANK_{iso1} (magenta) depicting the conformation of the long β-hairpin with its intervening helix α5 (blue), which is stabilized by helices α8 and η2 from a symmetry-related Ank domain (grey/pale cyan). The dotted lines indicate disordered regions flanking helix α5. The N- and C-termini are labeled.

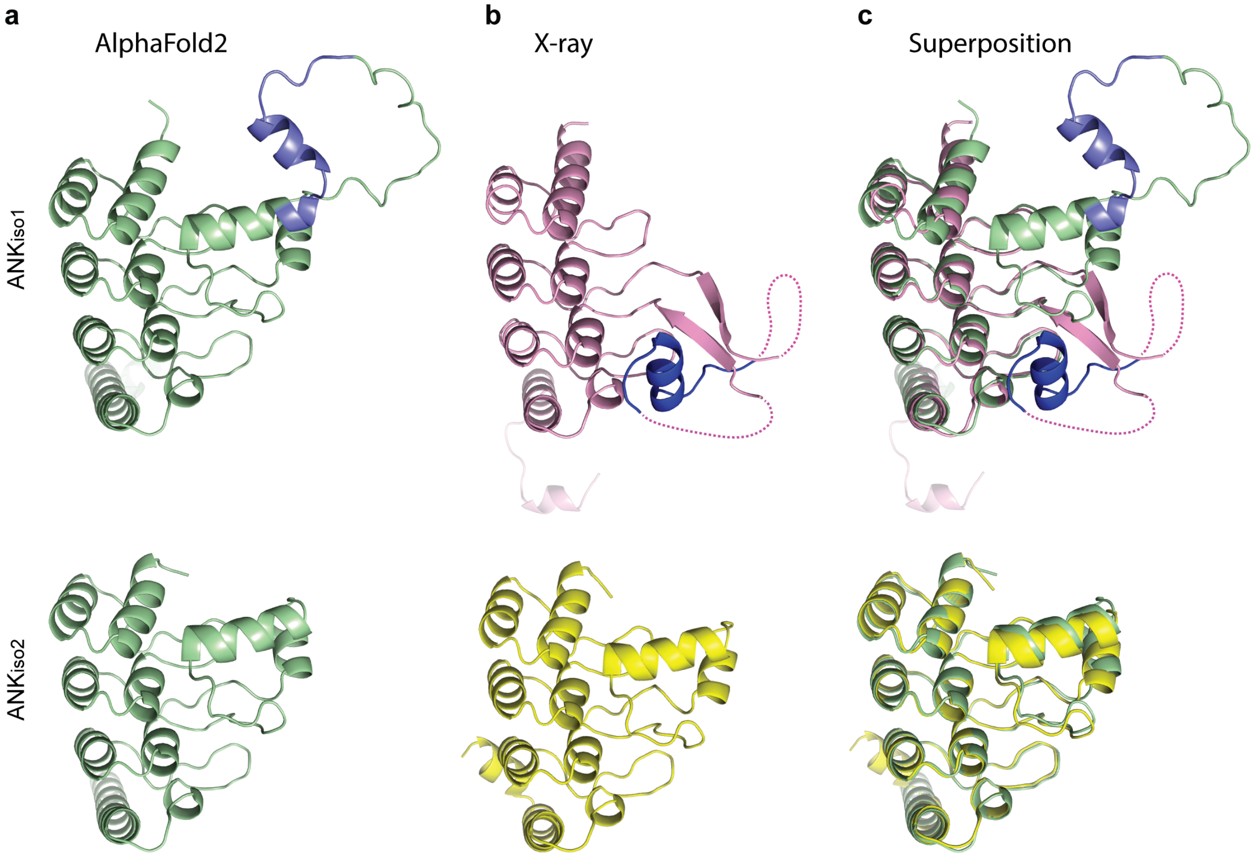

Supplementary Fig. 3: The X-ray structure of ANK_{iso1} differs from the *in silico* model predicted by AlphaFold2. **(a)** Ribbon diagram of the AlphaFold2[1] predicted structures for the Ank domain of human SKD3 isoform-1 (AF-Q9H078-F1) and isoform-2 (AF-G3RM06-F1). The Ank domain is shown in green with residues that include helix α5 as seen in the X-ray structure of ANK_{iso1} colored blue. **(b)** Ribbon diagram of the experimentally determined X-ray structures of human ANK_{iso1} (magenta) with helix α5 (blue) and human Ank_{iso2} (yellow). The dotted lines indicate disordered regions. **(c)** Superposition of the AlphaFold2 model and the X-ray structure of ANK_{iso1}, which superimpose with an RMSD of only 0.86 Å over 133 Cα atoms, and that of ANK_{iso2} and its AlphaFold2 prediction, which superimpose with an RMSD of only 0.57 Å over 146 Cα atoms. The long β-hairpin was excluded from the superimposition.

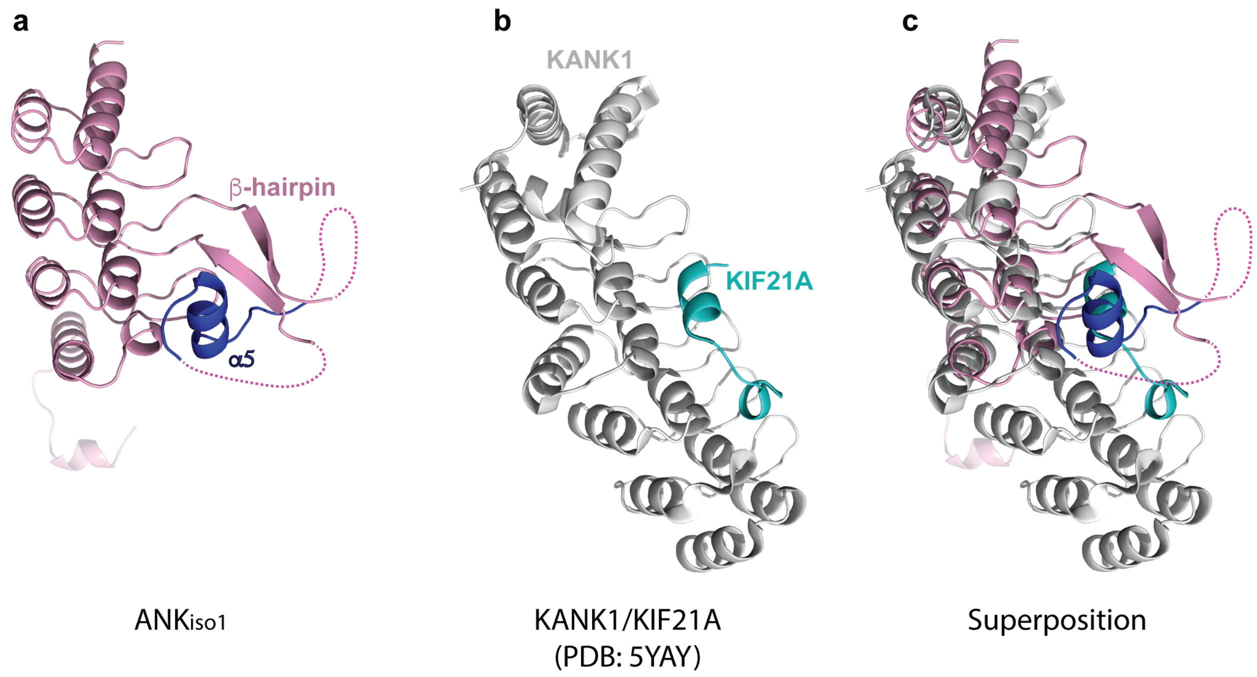

Supplementary Fig. 4: The X-ray structure of ANK$_{iso1}$ reveals a repressed conformation. **(a)** Ribbon diagram of the X-ray structure of human ANK$_{iso1}$ (magenta) with helix α5 colored blue. The dotted lines indicate disordered regions. **(b)** Ribbon diagram of the X-ray structure of an unrelated Ank domain-containing scaffold protein, KANK1 (grey), bound to a segment of its binding partner, KIF21A shown in cyan (PDB: 5YAY)[2]. The KANK1:KIF21A structure was identified bioinformatically[3] to be a close structural homolog of ANK$_{iso1}$. **(c)** Superposition of the Ank domains of SKD3 and KANK1 shows a close match. The KIF21A binding partner (cyan), which is partially helical, overlaps with helix α5 (blue) but differs in spatial orientation by a ~90° rotation. Considering this structural overlap, it is tempting to speculate that helix α5 may mimic a substrate interaction, which is displaced upon substrate binding.

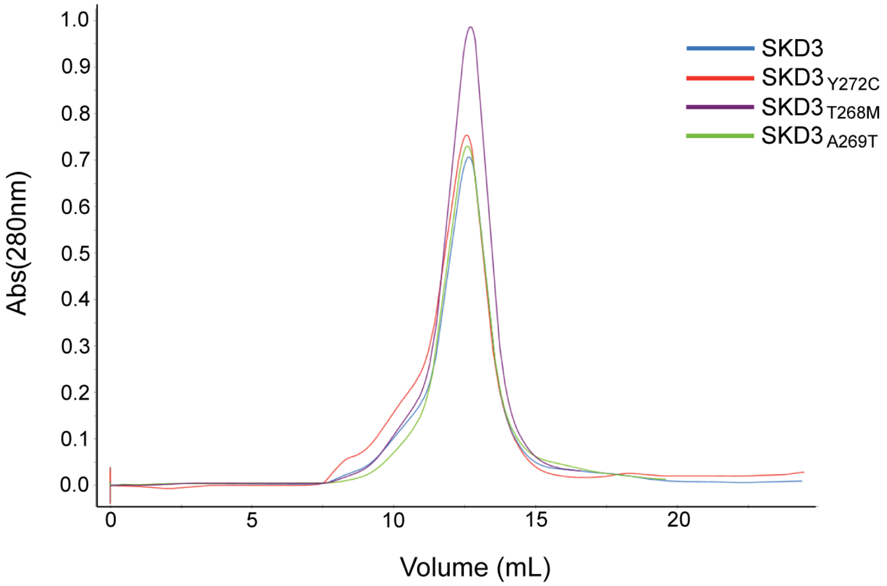

Supplementary Fig. 5: The oligomeric state of SKD3 and SKD3 Ank domain variants in solution. Overlayed SEC elution profiles for SKD3, $SKD3_{Y272C}$, $SKD3_{T268M}$, and $SKD3_{A269T}$ are shown, which are largely indistinguishable, indicating that Ank domain mutations do not alter the oligomeric state. All SKD3 oligomers were analyzed using a Superose 6 Increase 10/300 GL column.

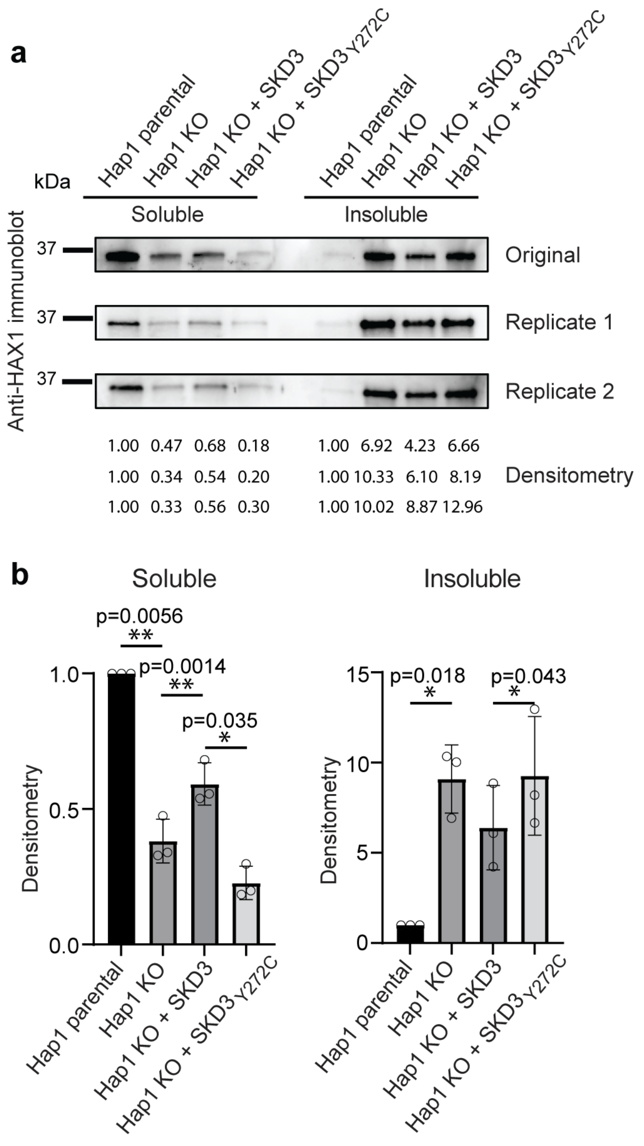

Supplementary Fig. 6: Quantitative densitometry analysis of HAX1 in Hap1 cells. **(a)** Western blot showing the relative solubility of HAX1 protein in wild-type (parental) and *SKD3* knockout (KO) Hap1 cells, and in *SKD3* KO Hap1 cells following viral transfection with wild-type *SKD3* or $SKD3_{Y272C}$. Densitometry measurements were performed for all experimental replicates (n = 3). **(b)** Relative quantitation of the changes induced by *SKD3* knockout and reintroduction of wild-type *SKD3* or $SKD3_{Y272C}$ mutant. Bar graphs show the mean densitometry measurement of all experimental replicates (n = 3) ± SD. P-values were calculated with the two-tailed paired Student's t-test (p<0.05 = *; p<0.01 = **).

Supplementary Table 1: Summary of data collection and refinement statistics.

	ANK_{iso1} (Se-Met)	ANK_{iso1}	ANK_{iso2}
**Data collection**			
Space group	$P\ 12_11$	$P\ 12_11$	$P\ 6_522$
Cell dimensions			
a, *b*, *c* (Å)	43.28, 61.13, 47.57	43.24, 61.09, 47.99	60.44, 60.44, 180.31
α, β, γ (°)	90.0, 106.3, 90.0	90.0, 106.3, 90.0	90.0, 90.0, 120.0
Wavelength (Å)	0.9794	0.9794	0.9792
Resolution (Å)	50-1.95 (1.97-1.95)	50-1.81 (1.88-1.81)	52.26-1.65 (1.68-1.65)
Unique Reflections	16,347	20,905	24,410
R_{sym} or R_{merge}	0.110 (0.508)	0.122 (0.578)	0.101 (0.532)
R_{pim}	0.056 (0.381)	0.059 (0.328)	0.024 (0.125)
$I / \sigma I$	11.2	9.9	9.0
Completeness (%)	92.3 (59.3)	99.1 (94.2)	100.0 (100.0)
Redundancy	3.7 (1.6)	4.7 (3.4)	18.3 (19.1)
**Refinement**			
Resolution (Å)		45.67-1.81	52.34-1.65
No. reflections		19,031/939	24,341/1,186
R_{work} / R_{free}		0.1633/0.1894	0.1991/0.2207
No. atoms		1,626	1,502
Protein		1,472	1,436
Water		154	57
B-factors		25.93	31.89
Protein		24.69	31.88
Water		37.67	31.59
RMS deviation			
Bond lengths (Å)		0.009	0.007
Bond angles (°)		0.920	0.863

Values in parentheses are for highest-resolution shell.

SUPPLEMENTARY REFERENCES

1. Jumper, J. et al. Highly accurate protein structure prediction with AlphaFold. *Nature* **596**, 583-589 (2021).
2. Pan, W. et al. Structural insights into ankyrin repeat-mediated recognition of the kinesin motor protein KIF21A by KANK1, a scaffold protein in focal adhesion. *J. Biol. Chem.* **293**, 1944-1956 (2018).
3. Zimmermann, L. et al. A completely reimplemented MPI bioinformatics toolkit with a new HHpred server at its core. *J. Mol. Biol.* **430**, 2237-2243 (2018).

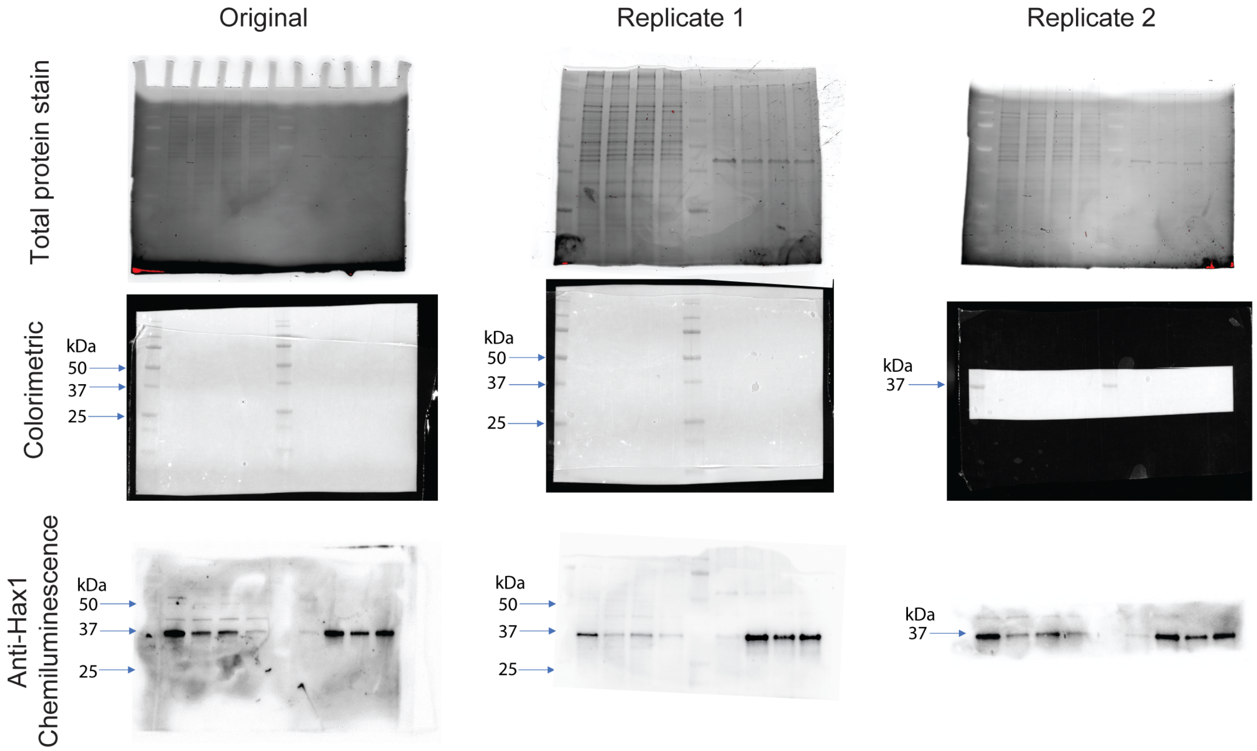

Supplementary source data for Supplementary Fig. 6.
