# Supplementary material for: Structural basis of impaired disaggregase function in the oxidation-sensitive SKD3 mutant causing 3-methylglutaconic aciduria
Source: Nat Commun. 2023 Apr 11;14:2028. doi: 10.1038/s41467-023-37657-9 (PMC10090083; doi:10.1038/s41467-023-37657-9)
Supplement: Supplementary file 2 — Reporting Summary [file 41467_2023_37657_MOESM2_ESM.pdf]

nature portfolio

Corresponding author(s): Francis T.F. Tsai

Last updated by author(s): Mar 17, 2023

Reporting Summary

Nature Portfolio wishes to improve the reproducibility of the work that we publish. This form provides structure for consistency and transparency in reporting. For further information on Nature Portfolio policies, see our Editorial Policies and the Editorial Policy Checklist.

Statistics

For all statistical analyses, confirm that the following items are present in the figure legend, table legend, main text, or Methods section.

n/a	Confirmed	
☐	☒	The exact sample size (*n*) for each experimental group/condition, given as a discrete number and unit of measurement
☐	☒	A statement on whether measurements were taken from distinct samples or whether the same sample was measured repeatedly
☐	☒	The statistical test(s) used AND whether they are one- or two-sided<br>*Only common tests should be described solely by name; describe more complex techniques in the Methods section.*
☒	☐	A description of all covariates tested
☐	☒	A description of any assumptions or corrections, such as tests of normality and adjustment for multiple comparisons
☐	☒	A full description of the statistical parameters including central tendency (e.g. means) or other basic estimates (e.g. regression coefficient) AND variation (e.g. standard deviation) or associated estimates of uncertainty (e.g. confidence intervals)
☐	☒	For null hypothesis testing, the test statistic (e.g. *F*, *t*, *r*) with confidence intervals, effect sizes, degrees of freedom and *P* value noted<br>*Give P values as exact values whenever suitable.*
☒	☐	For Bayesian analysis, information on the choice of priors and Markov chain Monte Carlo settings
☒	☐	For hierarchical and complex designs, identification of the appropriate level for tests and full reporting of outcomes
☒	☐	Estimates of effect sizes (e.g. Cohen's *d*, Pearson's *r*), indicating how they were calculated

Our web collection on statistics for biologists contains articles on many of the points above.

Software and code

Policy information about availability of computer code

Data collection	X-ray diffraction data were collected at the APS SBC ID19 beamline using the SBCCollect ver2 0.9.0.3 ID software. Biochemical measurements were performed using a Tecan Spark microplate reader using the Tecan SparkControl magellan standard v2.2 software .
Data analysis	X-ray diffraction data processing: HKL3000 Release 720 and DIALS. SAD phasing: MLPHARE. Density modification: DM. Initial model building: ARP/wARP. Structure determination: structure module of HKL3000 software suite. Model building and graphic display: COOT 0.8.9.2. Structure refinement: REFMAC 5.8.0222, PHENIX 1.16_3549 (ANKiso1), and PHENIX 1.20.1_4487 (ANKiso2). Sequence alignment: ESPript 3.0. Biochemical data analysis and plotting: GraphPad Prism 7.0 for Mac OS X. Densitometry measurements: FIJI suite of Image J (2.0.0-rc-69/1.52p; Java 1.8.0_172 [64-bit])

For manuscripts utilizing custom algorithms or software that are central to the research but not yet described in published literature, software must be made available to editors and reviewers. We strongly encourage code deposition in a community repository (e.g. GitHub). See the Nature Portfolio guidelines for submitting code & software for further information.

Data

Policy information about availability of data

All manuscripts must include a data availability statement. This statement should provide the following information, where applicable:

- Accession codes, unique identifiers, or web links for publicly available datasets
- A description of any restrictions on data availability
- For clinical datasets or third party data, please ensure that the statement adheres to our policy

Atomic coordinates and accompanying structure factors have been deposited with the RCSB with accession codes PDB: 8DEH (ANKiso1) and PDB: 8FDS (ANKiso2).

Human research participants

Policy information about studies involving human research participants and Sex and Gender in Research.

Reporting on sex and gender	Not applicable
Population characteristics	Not applicable
Recruitment	Not applicable
Ethics oversight	Not applicable

Note that full information on the approval of the study protocol must also be provided in the manuscript.

Field-specific reporting

Please select the one below that is the best fit for your research. If you are not sure, read the appropriate sections before making your selection.

☒ Life sciences ☐ Behavioural & social sciences ☐ Ecological, evolutionary & environmental sciences

For a reference copy of the document with all sections, see nature.com/documents/nr-reporting-summary-flat.pdf

Life sciences study design

All studies must disclose on these points even when the disclosure is negative.

Sample size	Three. No statistical method was used to predetermine sample size. Based upon accepted standards in the field, quantitative biochemical experiments and western blots were performed in at least three independent replicates. Detailed n number are provided in the figure legend. A representative western blot image from three replicates is presented, where applicable in the figure.
Data exclusions	None
Replication	Experiments were replicated more than or equal to three biological independent experiments for reproducibility of experimental findings. The number of replicates used for each data set is noted in the figure legends.
Randomization	Not applicable. This study did not involve experimental groups.
Blinding	Investigators were not blinded during the experiments. Blinding was not relevant for this study as no allocation of human/animal subjects was involved.

Reporting for specific materials, systems and methods

We require information from authors about some types of materials, experimental systems and methods used in many studies. Here, indicate whether each material, system or method listed is relevant to your study. If you are not sure if a list item applies to your research, read the appropriate section before selecting a response.

Materials & experimental systems

n/a	Involved in the study
☐	☒ Antibodies
☐	☒ Eukaryotic cell lines
☒	☐ Palaeontology and archaeology
☒	☐ Animals and other organisms
☒	☐ Clinical data
☒	☐ Dual use research of concern

Methods

n/a	Involved in the study
☒	☐ ChIP-seq
☒	☐ Flow cytometry
☒	☐ MRI-based neuroimaging

Antibodies

Antibodies used	Abcam, cat. #ab137613, anti-HAX1 antibody, lot #GR 3458141–1
Validation	This is a rabbit polyclonal anti-HAX1 antibody validated by the vendor for Western Blot applications against whole cell extract from different human cell lines.

Eukaryotic cell lines

Policy information about cell lines and Sex and Gender in Research

Cell line source(s)	Isogenic HAP1 parental (Cat. #C631) and HAP1 SKD3 knockout cells (Cat. #HZGHC007326c001) were directly acquired from Horizon Discovery Biosciences Ltd., Cambridge, UK.
Authentication	HAP1 parental and HAP1 SKD3 knockout cells were not further authenticated.
Mycoplasma contamination	Cells tested negative for mycoplasma contamination.
Commonly misidentified lines (See ICLAC register)	No commonly misidentified cell lines were used in the study.
